# The Effect of Music on aEEG Cyclicity in Preterm Neonates

**DOI:** 10.3390/children8030208

**Published:** 2021-03-09

**Authors:** Vito Giordano, Katharina Goeral, Leslie Schrage-Leitner, Angelika Berger, Monika Olischar

**Affiliations:** 1 Department of Pediatrics and Adolescent Medicine, Division of Neonatology, Pediatric Intensive Care and Neuropediatrics, Comprehensive Center for Pediatrics, Medical University of Vienna, 1090 Vienna, Austria; katharina.goeral@meduniwien.ac.at (K.G.); angelika.berger@meduniwien.ac.at (A.B.); monika.olischar@meduniwien.ac.at (M.O.); 2 Department of Music Therapy, University of Music and Performing Arts, Seilerstätte 26, 1010 Vienna, Austria; schrage-leitner@mdw.ac.at

**Keywords:** cyclicity, music, preterm, neonate, sleep–wake cycling

## Abstract

Several methods can be used in the neonatal intensive care unit (NICU) to reduce stress and optimize the quality of life during this period of hospitalization. Among these, music could play an important role. We investigated the effect of different kinds of music therapies on the brain activity of very preterm infants using amplitude-integrated EEG. Sixty-four patients were included and randomly assigned to three different groups: live music group, recorded music group, and control group. In both intervention groups, music was started after the appearance of the first quiet-sleep phase, with a subsequent duration of 20 min. Changes between the first and second quiet-sleep epochs were analyzed using the amplitude-integrated EEG. When looking at single parameters of the amplitude-integrated EEG trace, no differences could be found between the groups when comparing their first and second quiet-sleep phase regarding the parameters of change from baseline, quality of the quiet-sleep epoch, and duration. However, when looking at the total cyclicity score of the second quiet-sleep phase, a difference between both intervention groups and the control group could be found (live music therapy vs. control, *p* = 0.003; recorded music therapy vs. control, *p* = 0.006). Improvement within the first and second quiet-sleep epochs were detected in both music groups, but not in the control group. We concluded that our study added evidence of the beneficial effect of music on the amplitude-integrated EEG activity in preterm infants.

## 1. Background

Preterm infants are repeatedly exposed to painful procedures and stressful situations in the neonatal intensive care unit (NICU) [1,2,3]. Several methods can be used in the NICU to reduce stress and optimize the quality of life during this period of hospitalization [4]. Among these, music may play an important role [5].

Music represents the science and art of sound; it expresses and carries emotions having an impact on human physical conditions [6]. It is an auditory stimulus endowed with precise values such as harmony, melody, and rhythm, and is able to create a link between mind and body [7,8].

In fact, music can activate brain areas involved in movement, planning, attention, memory, and learning [7,8,9,10,11,12,13]; improve dopamine release associated with increased motivation, better mood, joy and pleasure [6]; enhance cognitive performance [7,8,9,11,13]; and help relieve pain [14].

Even if its therapeutic effect was already described in Greek mythology, only recently, after World War II, was music officially recognized as therapy [15,16]. According to a study by Kern and colleagues [15] investigating the role of music therapy worldwide, only a few resources have been identified in the field of pediatric intensive care. The factors behind this evidence could be several, including: the difficulty in implementing such therapy in this type of setting (e.g., hygienic reasons, noisy environment [17]); critical condition of the patients (e.g., requiring isolation); and financial priority of the actual medical system.

Despite these factors, music therapy shows promising results in the pediatric population, reducing anxiety induced by interventions, calming heart rate and respiratory rate, reducing stress levels before surgery, reducing the feeling of parental/peer separation due to isolation factors, and enhancing quality of life [18].

Evidence for the beneficial effect of music therapy is rising also for preterm babies [19,20,21]. Preterm babies, in fact, are already able to recognize auditory stimuli [19], as hearing is established as a functionally interactive sensation by the beginning of the third trimester [22].

In preterm neonates, music reduces heart rate (HR), improves feeding behavior, and leads to prolonged periods of quiet-alert state [23]. In late preterm infants, listening to recorded music leads to deeper quiet-sleep (QS) phases [24]. When studying adult electroencephalogram (EEG) activity, music produces an increased theta activity as a sign of relaxation [25]. Generally, music improves quality of sleep [26], while sleep in turn plays an important role in brain development and cognition [27,28,29,30] Recently, a clinical-practice protocol for music therapy in premature infants was published [31], and its effect on structural brain development was demonstrated [32]. The effect of music on the electrical cortical brain activity of very preterm infants is still underinvestigated. In addition, the impact of different kinds of music exposure (e.g., recorded music vs. live music) on the outcome of preterm infants remains unclear [20]. Therefore, the aim of this study was to investigate the effect of different kinds of music therapies on the brain activity of very preterm infants.

## 2. Methods

The present study was conducted in the two NICUs of the Medical University of Vienna (10-bed and 12-bed units) between September 2012 and December 2016, and was approved by the local ethics committee (EK 1634/2012). Preterm infants born before 32 weeks of gestation that were not sedated, without any history of perinatal asphyxia, signs of intraventricular hemorrhage, periventricular leukomalacia, or major cerebral malformations as seen on cranial ultrasound scans [33] were eligible for this study. Between 2012 and 2016, a total of 64 patients were included after parental informed consent. Patients were then randomly assigned to three different groups: a live music therapy (LMT) group, a recorded music therapy (RMT) group, and a control group.

The LMT intervention was performed by a qualified music therapist (LS) in the most possible standard way. The first step was getting into contact with the baby by gently touching the feet and caressing the head. In a second step, soft whispering and humming were used as vocal interactions. Melody was always adapted to the child’s responsiveness (breathing, facial expression, and gestures). Then, a children’s harp (pentatonic tuning) was added to the melody.

In the RMT group, preterm infants were exposed to “Brahms’ Lullaby” from the Australian-produced Music for Dreaming (Sound Impressions, Pty. Ltd.; www.musicfordreaming.com/index.htl (accessed on 4 January 2021). This recording was arranged to meet the recommendations for music as a stimulus in preterm infants [24]. “Brahms’ Lullaby” was also chosen because of its relaxing properties according to NICU music therapists and nurses [24,34]. RMT was played using speakers connected to an iPod (iPod classic A1238, Apple Inc.) and placed 30 cm from the head of the patient. Previously, in-ear sound level and noise environment were checked using an analog audio analyzer (Minilyzer ML1, NTi Audio AG). Music was played in accordance with the suggestion of the American Academy of Pediatrics [24].

Both interventions lasted for 20 min and started after the appearance of the first QS phase on amplitude-integrated electroencephalography (aEEG). The study procedure is also summarized in Figure S1.

Brain electrical activity was recorded using an aEEG (Olympic CFM™6000, Natus Medical Inc., San Carlos, CA, USA) by applying three gold electrodes placed at P3, P4, and FpZ.

**Figure 1:**
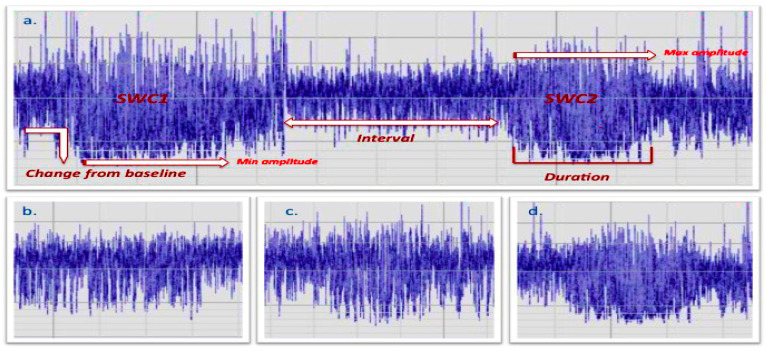
Representation of the aEEG sleep–wake cycling. (**a**) Representation of the aEEG epoch. Quite sleep epochs were defined as: (1) not well defined/fragmented (**b**); (2) defined but fragmented (**c**); or (3) defined and not fragmented (**d**).

The following aEEG characteristics were considered for statistical analysis: Burdjalov score [35], change from baseline (CBL) of the lower-margin in the transition between active sleep (AS) and quiet sleep (QS), quality/fragmentation of each QS epoch, and duration of each QS phase (Figure 1).

All aEEG characteristics were scored for the first and the second QS phase and the transition from one QS phase to the other in order to allow comparison before and after music exposure.

**Figure 2:**
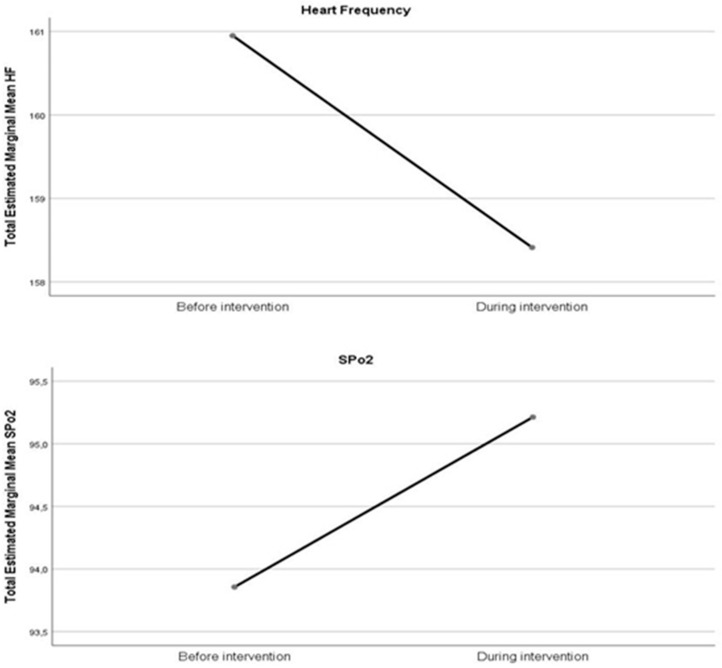
Change in vital signs before and during intervention.

CBL was scored as: (1) poor CBL; (2) modest CBL; or (3) evident CBL. Quality/fragmentation of QS epochs were defined as: (1) not well defined/fragmented (Figure 1b); (2) defined but fragmented (Figure 1c); or (3) defined and not fragmented (Figure 1d). This parameter was visually scored based on the aEEG trace; therefore, detailed information about raw-EEG activity such as tracé alternant and high-voltage slow pattern in QS epochs, low-voltage irregular pattern, and mixed pattern in AS [36] are not provided.

Based on our previous results [24], duration was defined as: (1) too short (<15 min duration); or (2) adequate (>15 min duration) [24]. Finally, a total cyclicity score (TCS) was obtained by calculating the sum of the three conditions mentioned above in order to integrate information between quality, fluctuation, and transition of different sleep stages. Mean HR and oxygen saturation (SpO2) were collected before and during intervention.

Statistical analysis was carried out using SPSS Statistics for Mac version 21.0 (IBM Corp., Armonk, NY, USA). Quantitative data are presented as means ± standard deviation (SD) or as median (Q1–Q3), while qualitative data are shown as counts and percentages. The chi-square test was used to compare percentage in the descriptive statistic section. The Kruskal–Wallis test was used to compare nonparametric differences between the groups. The Mann–Whitney U test was used as post hoc to look at specific differences between the groups (LMT vs. RMT; LMT vs. control; RMT vs. control). The Wilcoxon test for related samples was used to identify differences within the groups.

## 3. Results

A total of 64 patients were included in this study: 21 in the LMT group; 23 in the RMT group; and 20 in the control group. Five patients were excluded because of bad quality of the aEEG trace (two from the LMT group; two from the RMT group; and one from the control group). Patients were comparable for in-hospital demographic characteristics, including gestational age (GA) at birth and corrected GA at the assessment time point (Table 1).

Descriptive parameters about the aEEG are presented in Table S1.

The median Burdjalov score of the total aEEG trace was not different between the groups (LMT: 7 (6–10); RMT: 8 (6–10); control: 7 (5–9); *p* = 0.25) (Figure S2; Table 2)). In more detail, when looking at raw data, the distribution of the lower margin of the first QS phase (LMT: 3 (3–5); RMT: 4 (3–5); control: 3 (2–4); *p* = 0.123) and second QS phase (LMT: 3 (3–4); RMT: 4 (3–4); control: 3 (3–4); *p* = 0.082) was not statistically different between the groups. Similar results could be found with regard to the interval between the first and second QS period (LMT: 20 (15–50); RMT: 22 (19–35); control: 30 (20–40; *p* = 0.776). The median duration of the second QS epoch was significantly longer in both music groups compared to controls (*p* = 0.004) (Figure S3). When looking at scored parameters of the aEEG trace, no differences could be found in the first and second QS phase regarding CBL, quality, and duration between the groups. However, when looking at the total cyclicity score of the second QS phase, a difference between both intervention groups and the control group could be found (LMT vs. control, *p* = 0.003; RMT vs. control, *p* = 0.006) (Table 2, Figure S4).

When looking at analysis within the groups, an improvement within the first and second QS epoch could be noted in both music groups for the parameter change from baseline and total score (Table 3).

When looking at vital signs (Figure S5), even considering a general positive effect of music on vital signs (reduced heart rate and improved oxygenation) (Figure 2), no differences were found before and during music intervention (Table 3, Figure S6).

## 4. Discussion

The beneficial effect of recorded music therapy has already been demonstrated in studies using magnet resonance imaging. Improved white-matter maturation in acoustic radiations, external capsule/claustrum/extreme capsule, and uncinate fasciculus, as well as larger amygdala volume were described by Sa de Almeida and colleagues [37]. Promising results also have been reported in studies investigating the neurophysiological cortical activity of preterm and term-born infants [24,38]. To our knowledge, this is the first study investigating the effect of different kinds of music exposure in very preterm infants on their brain activity. According to our results, music intervention generally improved QS epochs of the aEEG trace. This was in accordance with what has been already described in the literature for different age groups [23,26,38,39].

Compared to our previous results [24], no differences were found regarding the lower margin of both QS phases; however, a more evident change from baseline was notable in the second QS epoch for both music groups. The discrepancies with our previous study could be related to the different GAs considered at the time-point of investigation. In fact, around term-age [24], aEEG activity is dominantly defined as continuous normal voltage (CNV), while a slower discontinuous normal voltage (DNV) activity can still be detectable in QS epochs [36,40,41,42,43]. On the contrary, preterm infants born prior to the 32nd week of gestation showed background activities that were still in a transitional stage of development, where both CNV and DNV activities were concomitantly dominant [36,41,42]. In addition, the bandwidth span was still high in AS phases when CNV activity usually is observed [35]. Therefore, we assumed that a more visually discernible QS epoch in the aEEG of premature infants could be better explained by a pronounced CBL in the transition between AS and QS, rather than a lower amplitude of the lower margin.

Similar to a recent study investigating the effect of RMT on 30 late preterm infants [38], an improvement in cyclicity was observed when referring to the total score of both music groups. However, in contrast to this study, we did not find any differences when looking at the Burdjalov score. The authors [38] concluded that patients who were exposed to music had a better Burdjalov score in AS epochs. However, even considering the different study designs (very preterm vs. late preterm infants; 20 min music exposure vs. 6 h music exposure), the Burdjalov score is usually calculated considering both AS and QS phases, and it is not possible to distinguish AS from wakefulness using aEEG. Previous studies indicated that AS played a very important role in promoting brain development [30]. However, there is also evidence that QS was essential for neuronal plasticity and synaptic remodeling [30]. In fact, more than the consideration of single parameters, it is the fluctuation between different sleep stages that represents a good index for later development. A more evident and detectable cyclicity has been shown to have a strong predictive value for neurodevelopmental outcome in preterm and term neonates [28,29]. According to our results, QS epochs after music exposure were longer and more discernable when looking at the total score compared to control.

Finally, even though we observed an improvement in HR and SpO2, this was not statistically significant. Literature published on this topic has reported divergent results. Most likely, as concluded by van der Heijden and colleagues in a systematic review, the age groups, time, and type of intervention make it difficult to draw strong conclusions regarding the effect of music on vital signs in preterm infants [44]. Finally, both types of music intervention had an impact on quality of QS in our study. However, even when considering the complex NICU setting [17,45], actual music therapy protocols [31] underline the psychosocial aspect of music therapy in the NICU by including and working together with the parents when possible. Haslbeck and colleagues [31] referred to the word “empowerment” when describing what could be achieved by music therapists and parents through communicative musicality, when looking at infants individual needs and responsiveness to music.

## 5. Limitation

This trial was conducted in a single center. Given the daily variability of clinical stability of preterm infants, the difficult setting, and maturational features, multiple recordings were not taken into consideration. Finally, EEG parameters were visually scored based on an aEEG trace; therefore, detailed information about raw EEG activity such as spectral analysis, tracé alternant, high-voltage slow pattern in QS epochs, low-voltage irregular pattern, and mixed pattern in AS were not provided

## 6. Conclusions

Even considering the difficult setting in which premature infants are hospitalized [17], evidence for the efficacy of music therapy on brain development is emerging [24,32], as are emerging standard protocols for music therapy in the NICU [31]. This study adds more evidence to the effect of music on aEEG activity in preterm infants, and suggests that music might contribute to an improved quality of hospitalization in these patients. Little is known about the effect of regular music exposure on short- and long-term neurodevelopmental outcome. Future studies will have to prove whether music offered during a stay in the NICU can have an impact on cognition and behavior by supporting brain maturation.

## Figures and Tables

**Table 1:** Demographic characteristics of patients.

	Live Music Therapy (*n* = 19)	Recorded Music Therapy (*n* = 21)	Control (*n* = 19)	*p*-Values
GA, weeks (mean ± SD)	28.16 ± 1.45	28.39 ± 2.07	27.51 ± 2.15	0.47
Corrected GA, weeks (mean ± SD)	30.27 ± 1.38	30.20 ± 1.53	29.39 ± 1.99	0.41
Male (*n*,%)	12 (63.2%)	12 (57.1%)	11 (57.1%)	0.91
Birth weight, grams (mean ± SD)	1050.10 ± 247.97	1104.00 ± 281.82	1037.15 ± 318.40	0.81
Umbilical cord pH (mean ± SD)	7.29 ± 0.95	7.30 ± 0.06	7.29 ± 0.91	0.77
Apgar 10 min (mean ± SD)	8.84 ± 0.50	9.00 ± 0.01	8.84 ± 0.68	0.33

Note: GA = gestational age.

**Table 2:** The aEEG analysis between the groups.

aEEG Parameters	Music Condition	Median (Q1–Q3)	Mean Rank	*p*-Value	Post-Hoch
Burdjalov score	LMT	7 (6–10)	30.66		
	RMT	8 (6–10)	32.55	0.25	N.S
	Control	7 (5–9)	26.53		
Change from baseline (first QS epoch)	LMT	2 (1–2)	29.26		
	RMT	2 (1–2,5)	31.50	0.85	N.S.
	Control	2 (1–2)	29.08		
Quality/fragmentation of QS (first QS epoch)	LMT	2 (2–2)	33.11		
	RMT	2 (2–2)	29.21	0.45	N.S.
	Control	2 (2–2)	27.76		
Duration scored (first QS epoch)	LMT	2 (1–2)	31.74		
	RMT	2 (1–2)	28.26	0.73	N.S.
	Control	2 (1–2)	30.18		
Total cyclicity score (first QS epoch)	LMT	6 (5–6)	32.45		
	RMT	5 (5–6,5)	29.81	0.67	N.S.
	Control	5 (4–6)	27.76		
Change from baseline (second QS epoch)	LMT	2 (2–3)	31.66		
	RMT	2 (2–3)	33.36	0.18	N.S.
	Control	2 (1–2)	24.63		
Quality/fragmentation of QS (second QS epoch)	LMT	2 (2–3)	31.95		
	RMT	2 (2–3)	31.93	0.36	N.S.
	Control	2 (2–2)	25.92		
Duration scored (second QS epoch)	LMT	2 (2–2)	32.34		
	RMT	2 (2–2)	32.79	0.76	N.S.
	Control	2 (1–2)	24.58		
Total cyclicity score (second QS epoch)	LMT	6 (6–7)	33.32		LMT vs Control *p* = 0.003
	RMT	6 (6–7)	34.57	0.02	RMT vs Control *p* = 0.006
	Control	6 (5–6)	21.63		LMT vs RMT *p* = 0.920

Note: QS = quiet sleep; Q1 = first quartile (25%); Q3 = third quartile (75%); N.S. = not significant. LMT = live music therapy. RMT = recorded music therapy.

**Table 3:** Change in vital signs and aEEG parameters within the groups before and after music intervention.

Live Music Therapy	Median (Q1–Q3)	Median (Q1–Q3)	*p*-Values
HR _Before vs. During_	164 (154–174)	158 (147–167)	0.129
Spo2 _Before vs. During_	96 (95–99)	96 (95–99)	0.156
Change from baseline score _QS epoch 1 vs. 2_	2 (1–2)	2 (2–3)	0.011
Quality/fragmentation of quiet sleep _QS epoch 1 vs. 2_	2 (2–2)	2 (2–3)	0.414
Duration score _QS epoch 1 vs. 2_	2 (1–2)	2 (2–2)	0.414
Total cyclicity score _QS epoch 1 vs. 2_	6 (5–6)	6 (6–7)	0.049
Recorded Music Therapy			
HR _Before vs. During_	157 (152–169)	158 (150–166)	0.545
SpO2 _Before vs. During_	92 (87–98)	94 (92–96)	0.127
Change from baseline score _QS epoch 1 vs. 2_	2 (1–2)	2 (2–3)	0.011
Quality/fragmentation of quiet sleep _QS epoch 1 vs. 2_	2 (2–2)	2 (2–3)	0.096
Duration score _QS epoch 1 vs. 2_	2 (1–2)	2 (2–2)	0.132
Total cyclicity score _QS epoch 1 vs. 2_	5 (5–6)	6 (6–7)	0.009
Control			
HR _Total_	157 (151–168)		
Spo2 _Total_	93 (91–97)		
Change from baseline score _QS epoch 1 vs. 2_	2 (1–2)	2 (1–2)	0.414
Quality/fragmentation of quiet sleep _QS epoch 1 vs. 2_	2 (2–2)	2 (2–2)	0.705
Duration score _QS epoch 1 vs. 2_	2 (1–2)	2 (1–2)	0.480
Total cyclicity score _QS epoch 1 vs. 2_	6 (4–6)	6 (5–6)	0.855

Note: HR = heart rate; SpO2 = oxygen saturation; QS = quiet sleep.

## Data Availability

All data requests should be submitted to the corresponding author for consideration. Access to anonymized data may be granted following review.

## Supplementary Materials

The following are available online at https://www.mdpi.com/2227-9067/8/3/208/s1, Figure S1: Iconographic of study procedure; Figure S2: Burdjalov score; Figure S3: Duration of the second quite sleep epoch; Figure S4: Related-sample Wilcoxon signed rank test; Figure S5: Distribution of hearth frequencies values; Figure S6: Box-plot of hearth rate frequency.

## Abbreviations

aEEG: amplitude-integrated electroencephalography; AS: active sleep; CBL: change from baseline; CNV: continuous normal voltage; GA: gestational age; HR: heart rate; LMT: live music therapy; NICU: neonatal intensive care unit; QS: quiet sleep; RMT: recorded music therapy; SpO2: oxygen saturation; TCS: total cyclicity score.
