# Supplementary material for: The Effect of Music on aEEG Cyclicity in Preterm Neonates
Source: Children (Basel). 2021 Mar 9;8(3):208. doi: 10.3390/children8030208 (PMC8000223; doi:10.3390/children8030208)
Supplement: Supplementary file 1 [file children-08-00208-s001.pdf]

Supplementary:

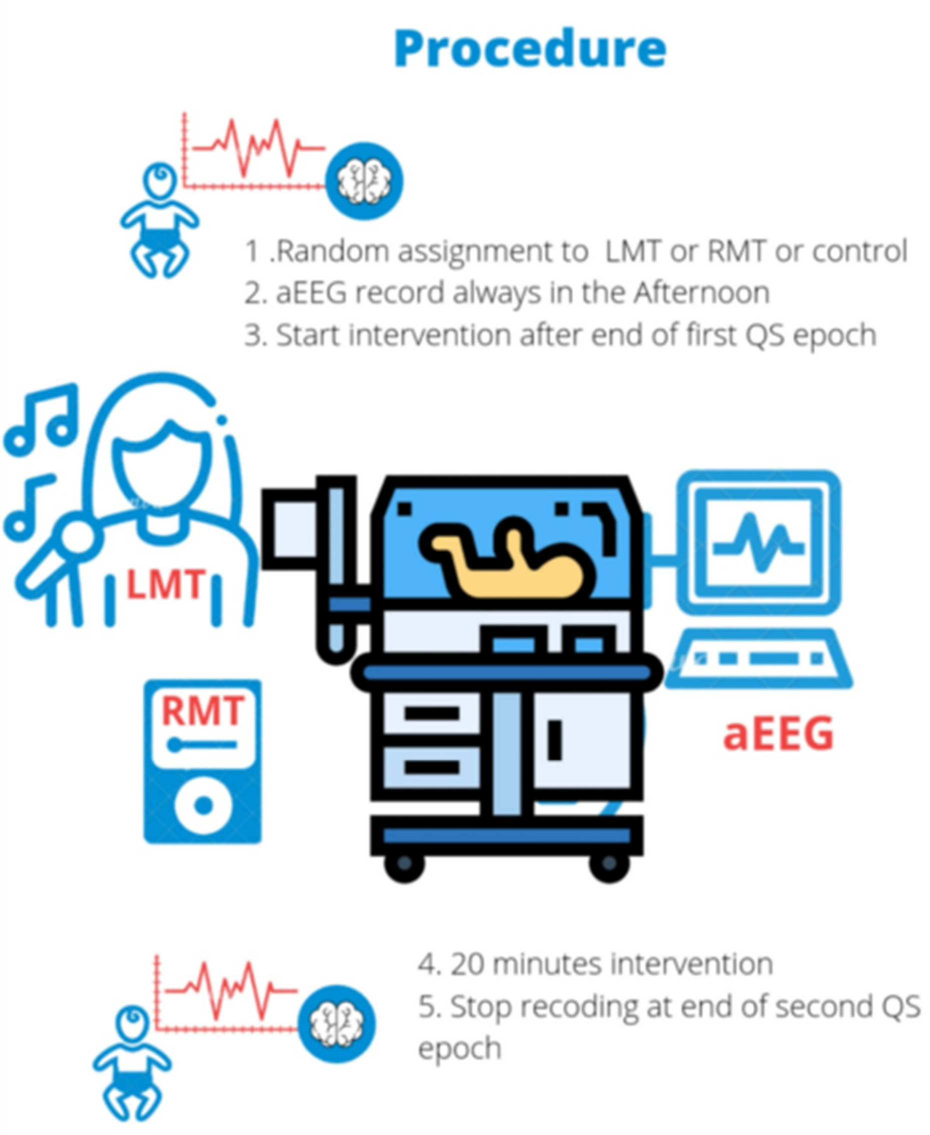

Figure S1. Iconographic of study procedure.

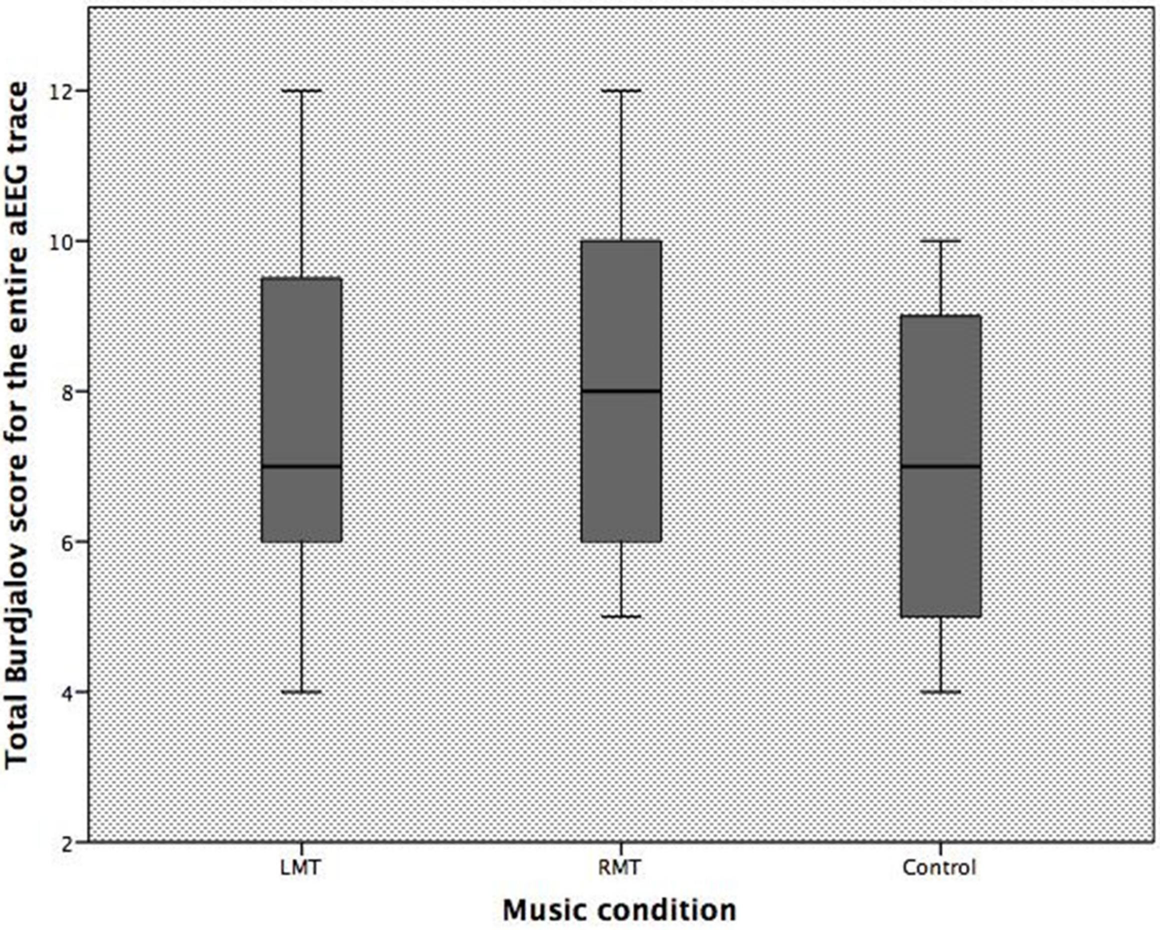

Figure S2. Burdjalov score.

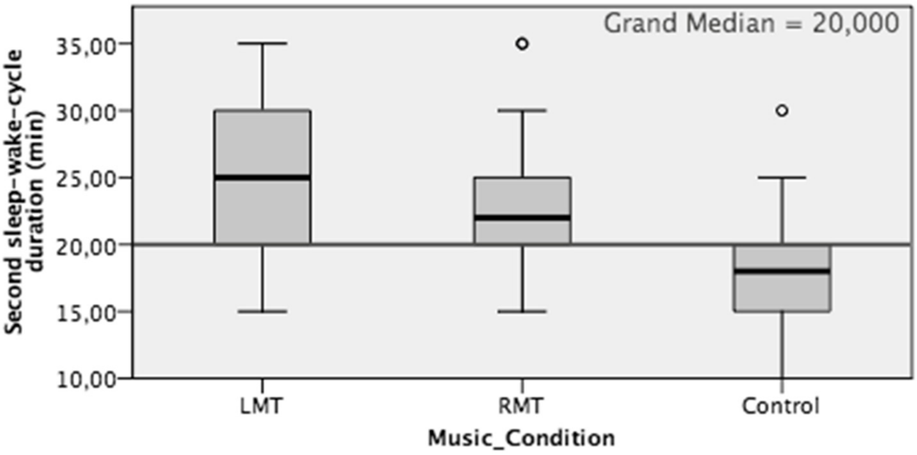

Figure S3. Duration of the second quite sleep epoch.

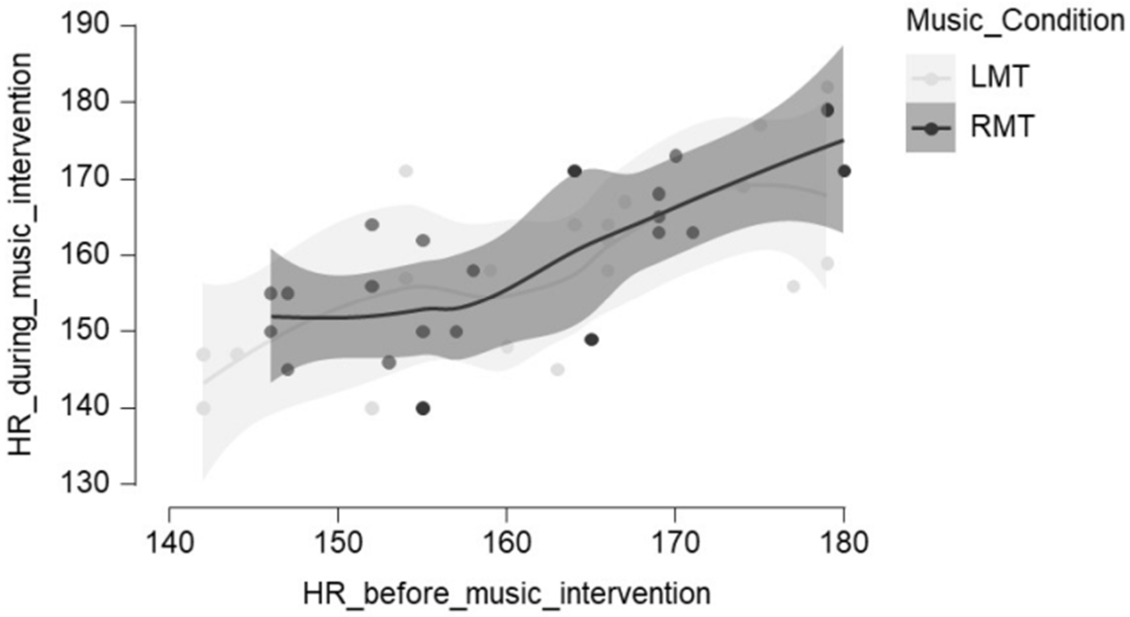

Figure S4. Related-sample Wilcoxon signed rank test.

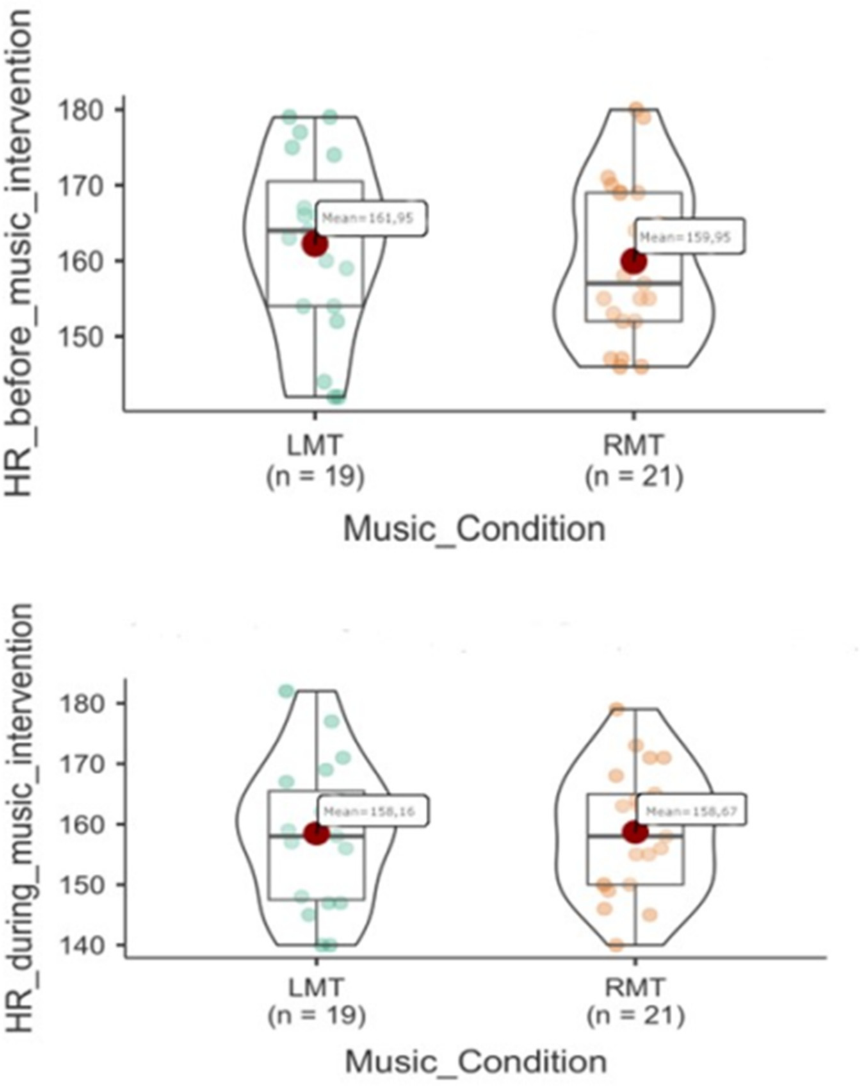

Figure S5. Distribution of hearth frequencies values.

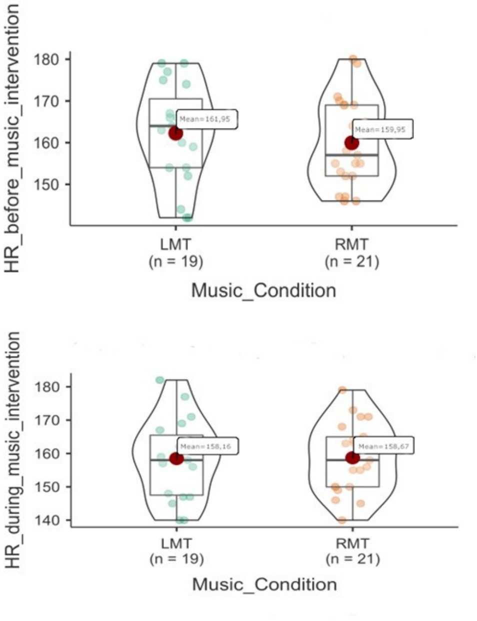

Figure S6. Box-plot of hearth rate frequency.
